# Innovations in phenotyping of mouse models in the German Mouse Clinic

**DOI:** 10.1007/s00335-012-9415-1

**Published:** 2012-08-29

**Authors:** Helmut Fuchs, Valérie Gailus-Durner, Susanne Neschen, Thure Adler, Luciana Caminha Afonso, Juan Antonio Aguilar-Pimentel, Lore Becker, Alexander Bohla, Julia Calzada-Wack, Christian Cohrs, Anna Dewert, Barbara Fridrich, Lillian Garrett, Lisa Glasl, Alexander Götz, Wolfgang Hans, Sabine M. Hölter, Marion Horsch, Anja Hurt, Eva Janas, Dirk Janik, Melanie Kahle, Martin Kistler, Tanja Klein-Rodewald, Christoph Lengger, Tonia Ludwig, Holger Maier, Susan Marschall, Kateryna Micklich, Gabriele Möller, Beatrix Naton, Cornelia Prehn, Oliver Puk, Ildikó Rácz, Michael Räß, Birgit Rathkolb, Jan Rozman, Markus Scheerer, Evelyn Schiller, Anja Schrewe, Ralph Steinkamp, Claudia Stöger, Minxuan Sun, Wilfried Szymczak, Irina Treise, Ingrid Liliana Vargas Panesso, Alexandra M. Vernaleken, Monja Willershäuser, Annemarie Wolff-Muscate, Ramona Zeh, Jerzy Adamski, Johannes Beckers, Raffi Bekeredjian, Dirk H. Busch, Oliver Eickelberg, Jack Favor, Jochen Graw, Heinz Höfler, Christoph Höschen, Hugo Katus, Martin Klingenspor, Thomas Klopstock, Frauke Neff, Markus Ollert, Holger Schulz, Tobias Stöger, Eckhard Wolf, Wolfgang Wurst, Ali Önder Yildirim, Andreas Zimmer, Martin Hrabě de Angelis

**Affiliations:** 1 German Mouse Clinic, Institute of Experimental Genetics, Helmholtz Zentrum München, German Research Center for Environmental Health (GmbH), Ingolstädter Landstraße 1, 85764 Neuherberg/Munich, Germany; 2 Molecular Nutritional Medicine, Else Kröner-Fresenius Center, TUM, Gregor-Mendel-Straße 2, 85350 Freising-Weihenstephan, Germany; 3 Chair for Molecular Animal Breeding and Biotechnology, Gene Center, Ludwig-Maximilians-Universität München, Feodor Lynen-Straße 25, 81377 Munich, Germany; 4 Institute for Medical Microbiology, Immunology, and Hygiene, TUM, Trogerstraße 30, 81675 Munich, Germany; 5 Clinical Research Division of Molecular and Clinical Allergotoxicology, Department of Dermatology and Allergy, TUM, Biedersteiner Straße 29, 80802 Munich, Germany; 6 Division of Environmental Dermatology and Allergy TUM/Helmholtz Zentrum München, German Research Center for Environmental Health (GmbH), Ingolstädter Landstraße 1, 85764 Neuherberg/Munich, Germany; 7 Department of Neurology, Friedrich-Baur-Institut, Ludwig-Maximilians-Universität München, Ziemssenstraße 1a, 80336 Munich, Germany; 8 Institute of Pathology, Helmholtz Zentrum München, German Research Center for Environmental Health (GmbH), Ingolstädter Landstraße 1, 85764 Neuherberg/Munich, Germany; 9 Institute of Developmental Genetics, Helmholtz Zentrum München, German Research Center for Environmental Health (GmbH), Ingolstädter Landstraße 1, 85764 Neuherberg/Munich, Germany; 10 Abteilung Medizinische Strahlenphysik und Diagnostik, Helmholtz Zentrum München, German Research Center for Environmental Health (GmbH), Ingolstädter Landstraße 1, 85764 Neuherberg/Munich, Germany; 11 Comprehensive Pneumology Center, Institute of Lung Biology and Disease, Helmholtz Zentrum München, German Research Center for Environmental Health (GmbH), Ingolstädter Landstraße 1, 85764 Neuherberg/Munich, Germany; 12 Institute of Molecular Psychiatry, University of Bonn, Sigmund-Freud-Straße 25, 53105 Bonn, Germany; 13 Chair of Experimental Genetics, Center of Life and Food Sciences Weihenstephan, TUM, 85350 Freising-Weihenstephan, Germany; 14 Division of Cardiology, Department of Medicine III, University of Heidelberg, Otto-Meyerhof-Zentrum, Im Neuenheimer Feld 350, 69120 Heidelberg, Germany; 15 Institute of Pathology, TUM, Ismaningerstraße 22, 81675 Munich, Germany; 16 Institute of Human Genetics, Helmholtz Zentrum München, German Research Center for Environmental Health (GmbH), Ingolstädter Landstraße 1, 85764 Neuherberg/Munich, Germany; 17 Chair of Developmental Genetics, TUM, Am Hochanger 8, 85350 Freising-Weihenstephan, Germany; 18 Institute of Epidemiology 1, Helmholtz Zentrum München, German Research Center for Environmental Health (GmbH), Ingolstädter Landstraße 1, 85764 Neuherberg/Munich, Germany; 19 Max-Planck-Institute of Psychiatry, Kraepelinstraße 2-10, 80804 Munich, Germany; 20 Deutsches Zentrum für Neurodegenerative Erkrankungen e. V. (DZNE) Site Munich, Schillerstraße 44, 80336 Munich, Germany; 21 Member of German Center for Diabetes Research (DZD), 85764 Neuherberg, Germany

## Abstract

Under the label of the German Mouse Clinic (GMC), a concept has been developed and implemented that allows the better understanding of human diseases on the pathophysiological and molecular level. This includes better understanding of the crosstalk between different organs, pleiotropy of genes, and the systemic impact of envirotypes and drugs. In the GMC, experts from various fields of mouse genetics and physiology, in close collaboration with clinicians, work side by side under one roof. The GMC is an open-access platform for the scientific community by providing phenotypic analysis in bilateral collaborations (“bottom-up projects”) and as a partner and driver in international large-scale biology projects (“top-down projects”). Furthermore, technology development is a major topic in the GMC. Innovative techniques for primary and secondary screens are developed and implemented into the phenotyping pipelines (e.g., detection of volatile organic compounds, VOCs).

## German Mouse Clinic I: systemic phenotyping

**Fig. 1:**
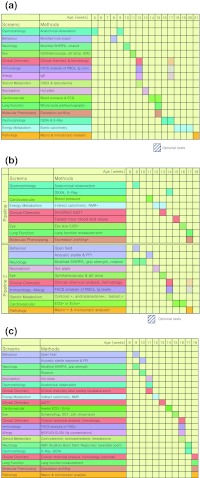
**a** Single pipeline that was used for phenotyping in the beginning phase of the German Mouse Clinic. **b** Two-pipeline system for first-line phenotyping that was introduced to contribute to the phenotyping activities within EUMODIC. The pipelines cover all tests that are mandatory for EMPReSSslim but include additional tests specific for the GMC (labeled by *asterisks*). **c** Modern single pipeline: new version of a single phenotyping pipeline that will be applied for phenotyping in bottom-up projects within Infrafrontier

The German Mouse Clinic (GMC, www.mouseclinic.de; Gailus-Durner et al. 2005; Fuchs et al. 2011) started in 2001 with the systemic analysis of mutant mouse lines as model systems for human diseases. The aim of the standardized phenotyping program in the GMC is to obtain a characterization of mutant mouse lines for most medically relevant areas in order to assign the mutant line to a certain disease or syndrome and to obtain insight into the diseases. The phenotyping of mouse lines in the German Mouse Clinic is divided into a primary screen, for an almost comprehensive and standardized characterization in the fields allergy, behavior, cardiovascular, clinical chemistry, diabetes, dysmorphology, bone and cartilage, energy metabolism, steroids, eye and vision, immunology, lung function, molecular phenotyping, neurology, nociception, and pathology, as well as secondary and tertiary in-depth and hypothesis-driven screens with more sophisticated phenotyping technologies. In the primary screen, a cohort of male and female mutants and controls (either littermate or stock breeding controls) is analyzed in a portfolio of tests that cover the whole spectrum of the phenotyping platform. Following a standardized pipeline of tests, the mice are examined in all modules of the GMC. The phenotyping pipeline plays a central role for the phenotyping activities in mouse clinics. It has to be kept up to date as new technologies become available and should be implemented into the phenotyping procedure. Logistics and animal welfare also have to be considered for the pipeline design. Examples for pipelines and the tests therein are shown in Fig. 1a–c.

The GMC is organized as a “multi-institutional” structure where biologists, physicians, veterinarians, computer scientists, and statisticians work together under the same (physical) roof for the phenotypic analysis of mouse models. For each of the aforementioned research areas, a module with a specialized scientist is in place to conduct the phenotypic analysis. Many of the modules are headed by or associated with clinicians who cover the disease area and have direct access to patients. In this case, a postdoctorate of the lab runs the GMC module and stays in regular contact with the home lab. Thus, a direct connection from the mouse model to the human disease is guaranteed. This special structure of the GMC with experts from various disciplines also ensures that the screens stay innovative and up-to-date.

One characteristic of the GMC is that on the one hand it contributes to large-scale “top-down” international mouse phenotyping initiatives like EUMODIC (www.eumodic.org) or the International Mouse Phenotyping Consortium (IMPC, www.mousephenotype.org), and on the other hand it is open for bilateral collaborations via “bottom-up” projects. Requests for phenotyping in “bottom-up” projects can be submitted via the GMC web page www.mouseclinic.de. All projects aim to lead to a joint publication of the data. For bottom-up projects, a direct import of complete cohorts that are ready for phenotyping is possible under adherence to specific sanitary guidelines. Sophisticated logistics have been put in place to afford this kind of effort in a direct collaboration with a mouse-providing institute. To fulfill all sanitary needs, special housing conditions with complete IVC racks and close collaboration with the veterinarian department of the Helmholtz Zentrum München is in place. For the logistic organization of all steps that are involved, an experienced administrative team coordinates the interplay of all procedures.

In the past decade, more than 250 mutant mouse lines were analyzed in the phenotyping screen of the GMC. In more than 90 % of the analyzed mutant lines, new or additional phenotypes were found. The results of the phenotyping activities in the GMC can be classified into three categories, discussed below.

### Discovery of unknown gene functions and pleiotropic effects

The precise function of many genes is still unknown and has to be elucidated. The phenotypic characterization of a mutant mouse line can deliver information about the functions and existing pleiotropic effects and will help to generate working hypotheses for the next steps. A very prominent example of where the primary screen of the GMC contributed to the elucidation of a gene’s function is *FoxP2*. *FoxP2* is a ubiquitously expressed transcription factor that has been highly conserved during human evolution. *FoxP2* sequences of human and chimpanzee differ by only two amino acids. Even the mouse genomic sequence differs by only one amino acid substitution from chimpanzee. Two functional copies of *FOXP2* are needed for humans to develop normal speech and language (Lai et al. 2001; MacDermot et al. 2005; Vargha-Khadem et al. 1998). Adult mice that carried a human version of the *Foxp2* sequence were analyzed in the systemic primary screen at the GMC and were compared to mice lacking one functional copy of the gene. It was found that the *Foxp2* ^hum^ allele specifically affects exploratory behavior (tested in two independently generated lines); no further phenotypes in any other parameter set or organ were detected (Enard et al. 2009). Furthermore, medium spiny neurons have increased dendrite lengths and increased synaptic plasticity. However, mice carrying one nonfunctional copy of *Foxp2* showed opposite effects, less neophobic behavior, and impaired motor coordination (Enard et al. 2009). The results of the systemic phenotyping at the GMC verified that the altered exploratory behavior in *Foxp2* ^hum^ mice is not caused by a loss of function of the gene product caused by the altered gene sequence or a general unhealthy condition, which might not have been proven by a screening for aberrant behavioral phenotypes alone.

Missing-in-metastasis (MIM/MTSS1) is a tissue-specific regulator of actin and plasma membrane dynamics mediating cell polarity and motility (Yu et al. 2011). Data from patients suffering from several cancer types indicate that the level of MTSS1 serves as a prognostic marker. MTSS1 might act as a putative tumor suppressor in human bladder cancer where MTSS1 could not be detected by immunohistochemistry (Du et al. 2011). In patients suffering from esophageal squamous cell carcinoma, the levels of MTSS1 transcript correlated with tumor-grade lymph node metastasis and overall survival; thereby, patients with high levels had a favorable prognosis (Xie et al. 2011) and hepatocellular carcinoma (Fan et al. 2012). However, the opposite was observed in tissue samples from colorectal cancer patients: high MTSS1 protein levels correlate with poor differentiation and prognosis (Wang et al. 2011). The dual function as tumor suppressor or tumor-promoting factor depending on the cancer type had to be elucidated. In a first step we analyzed the function of MIM in healthy mice. Phenotypic analysis of young and aged homozygous MIM-null mice displayed a severe urinary concentration defect in aged mice as polyuria and renal electrolyte wasting in the clinical chemistry screen. Elevated plasma alkaline phosphatase, suggesting high bone turnover, went together with reduced bone content in dual-energy X-ray absorptiometry (DXA). Transmission electron microscopy (TEM) analysis of the kidney of aged animals revealed that these functional alterations are caused by compromised integrity of kidney epithelia intercellular junctions. The data of the GMC demonstrated a new function of MIM in modulating the actin cytoskeleton–plasma membrane interaction to promote the maintenance of cell–cell contacts in kidney epithelia (Saarikangas et al. 2011).

### Models for human diseases

Since experts from different fields analyze the mutant mouse lines at the GMC, systemic effects of the mutation on the whole organism can be detected, which might remain undiscovered by investigations that are focused on a specific field of interest. These findings add valuable information to the overall picture and allow the study of the pathophysiological processes that cause the disease.

The transcription factor *Pitx3* plays an important role in dopamine neuron development as well as in survival (Li et al. 2009). It has recently been shown in epidemiological studies (Bergman et al. 2010; Liu et al. 2011; Haubenberger et al. 2011) that polymorphisms in the *Pitx3* gene are associated with sporadic and early-onset forms of Parkinson’s disease (PD), which account for approximately 90 % of PD cases. Analyzing the mutant mouse line Eyeless, which carries a homozygous point mutation in the coding region of the *Pitx3* gene (Rosemann et al. 2010), revealed defects that are also observed in PD patients such as motor impairments and loss of dopamine neurons of the substantia nigra. Additionally, the mutation affected until then unknown pathways and functions such as hematopoiesis and nociceptive behavior; this opens up new avenues for further investigations of the underlying mechanisms and diagnosis of early-onset PD.

Human renal diseases are often characterized by multiple symptoms. Uromodulin or Tamm–Horsfall protein is the most abundant protein in human urine under physiological conditions. Uromodulin storage disease is a dominantly inherited medical condition associated with low urine osmolality, hyperuricemia, and progressive renal failure, ultimately resulting in end-stage renal disease (Lhotta 2010; Vyletal et al. 2010). Homozygous adult animals of the mutant mouse line *Umod* ^A227T^ share most of the clinical symptoms concerning kidney function with affected human patients, i.e., elevated plasma urea levels, defects in the urine concentration mechanism, and excretion of urinary solutes. Hyperuricemia was only moderately present in mutant mice which might be explained by preserved uricase activity in mice. The systemic phenotypic characterization of the mutant mouse revealed for the first time additional effects on energy (slight hypometabolism and reduced body temperature) and bone metabolism (osteopenia). This illustrates the physiological interactions and systemic consequences of renal defects such as the long-term effects of hypercalciuria on bone mineral density (Kemter et al. 2009).

The mouse model of human type I Bartter syndrome *Slc12a1* ^I299F^ is the first described viable and fertile mouse model of this disease, displaying most symptoms seen in human patients suffering from antenatal Bartter syndrome. The symptoms are more severe for human patients, who are homozygous carriers of *SLC12A1* mutations already suffering from polyuria during gestation leading to prenatal polyhydramnios, whereas the mouse model did not show pathological changes during gestation or suckling periods. Nevertheless, 3-month-old homozygous *Slc12a1* ^I299F^ animals present most of the typical symptoms such as polyuria, polydipsia, and impaired ability to concentrate urine. Furthermore, the long-term effect of hypercalciuria on bone mineral density was investigated in 9-month-old animals. Indications for osteopenia could be observed. This mouse line of late-onset manifestation of type I Bartter syndrome might be a valuable model in which to test new therapeutic strategies for salt-losing tubulopathies (Kemter et al. 2009, 2010)

### Systemic phenotyping used for target validation and modeling therapeutic intervention

Screening of mutant mouse lines generated by a knockout or knockdown strategy allows the detection of potential side effects of drug targets. For a long time it has been proposed that oxidative stress is a candidate mechanism in ischemic stroke leading to neuronal death. Among the NADPH oxidases, NOX4 was identified as being induced during ischemic stroke in mice and humans. Adult *Nox4* knockout mice were analyzed in a comprehensive phenotype screen under standard conditions without detecting abnormalities that would suggest potential side effects of a drug decreasing NOX4 function. However, after both transient and permanent cerebral ischemia, these mice were largely protected from oxidative stress, blood–brain barrier leakage, and neuronal apoptosis such as in wild-type mice after application of the NADPH oxidase inhibitor VAS2870 (Kleinschnitz et al. 2010).

Other examples for modeling time-limited medication are approaches that use gene silencing, e.g., by sequence-specific RNA interference (RNAi). The most attractive target genes are oncogenes such as the critical regulator of mitosis, polo-like kinase 1 (Plk1). Homozygous *Plk1*-null mice are embryonic lethal. However, if the protein synthesis is reduced in adult mice by the inducible expression of small hairpin RNA over a period of 6 weeks, no major structural or functional anomalies are identified by systemic phenotypic screening. This observation is diametrically opposed to the role of Plk1 in cancer cells of varying origins which, following the inhibition of Plk1, quickly cease dividing and enter apoptosis. In contrast to tumor cells, healthy primary cells are dependent only on Plk1 expression to a minimal degree (Raab et al. 2011).

The data from finalized phenotyping projects is available for download on the web page of the German Mouse Clinic under http://www.mouseclinic.de/phenomap/phenomap.html. The data are displayed as a heat plot, and, in addition, a complete phenotyping report can be downloaded.

## German Mouse Clinic II: genome meets environment: envirotype simulation

**Fig. 2:**
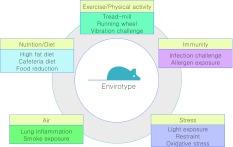
The challenge platform within the German Mouse Clinic II

For the analysis of complex human diseases, two components—the genetic predisposition and environmental factors such as lifestyle and aging—have to be considered. Beckers et al. (2009) described this relationship as a triangle of genotype, envirotype, and phenotype, each of which interacts with the other. As for understanding the involved biological pathways, the interplay between genetic and environmental factors has to be investigated. Mouse model systems can help us to learn more about these interactions by the application of challenge experiments. In a challenged situation, mutant mouse lines may show phenotypes that would remain hidden under unchallenged conditions. In the GMC, a challenge platform was established to analyze mutant mouse lines under altered environmental situations (Fig. 2). The challenge experiments are designed to simulate the major environmental factors that influence human health: infection, diet, air, activity, and stress. For each of these factors, challenge tests ranging from acute to chronic impact were developed: by feeding mice with different diets (such as a high-fat diet or a cafeteria diet), reactions to the change of physiological parameters can be provoked. For the air/lung system, instillation (acute reaction) or inhalation (chronic reaction) of diesel particles are applied. Immune challenges comprise an ovalbumin (OVA) challenge and infection with *Listeria monocytogenes*. For the stimulation of stress reactions, mice are kept under restraint, exposed to light, or oxidative stress is provoked by chemicals. In an activity platform, mice are allowed to run on a treadmill or on running wheels; in addition, muscle contractions are stimulated by vibration plates. For readout, the complete phenotyping portfolio of the GMC can be consulted. Tailor-made pipelines are designed for each single experiment to yield the maximum information about the mutant mouse line.

A first example, where the nutrition challenge platform was applied, is the mutant mouse line Eps8. Mice deficient for the *Eps8* gene displayed reduced body weight. This was not caused by reduced food intake, even when fed a high-fat diet. *Eps8* knockout mice showed partial resistance to age- or diet-induced obesity, overall improved metabolic status, and increased life span. Since the feces of *Eps8* knockout mice had higher energy content than those of normal mice, on a normal diet and on a high-fat diet, it was possible to identify the mechanisms behind this phenotype. The calorie restriction-like phenotype correlated with a significant reduction in intestinal fat absorption, presumably due to a 25 % reduction in intestinal microvilli length. An analysis of the subcellular localization of Eps8 in intestinal cells suggested that Eps8 is localized in intestinal microvilli. Since microvilli serve to enlarge the absorptive surface of the intestine, their reduction in *Eps8* knockout mice explains the improved metabolic status of *Eps8* knockout mice. This assumption is supported by alterations in the gene expression profile of livers from *Eps8* knockout mice that were comparable to those typical for conditions of calorie restriction (Tocchetti et al. 2010).

Another option of the envirotype challenge platform of the German Mouse Clinic is to combine the strength of different challenge tests. A setup with combined alterations in the environment reflects an approach to the complex environmental situations in real human life. Götz et al. (2011) published a study in which the effects of a diet and a lung challenge were combined to find out whether there are connections between adipose tissue and susceptibility to chronic lung diseases such as emphysema and asthma. Mice fed an energy-dense carbohydrate-rich diet or a high-fat diet were compared to mice on a low-fat diet. Bronchoalveolar lavage and blood samples were taken after instillation of intratracheal carbon nanoparticles. The study concluded that extended feeding periods are necessary to generate increased susceptibility to particle-induced lung inflammation, although the diet challenge already was efficient in driving proinflammatory systemic events.

## German Mouse Clinic III: systemic analysis of compounds and drugs

Applied research that involves the use of animal models of diseases or particular disease conditions is usually an early stage in the process of drug discovery. From an ethical point of view, drug tests in clinical trials should not be conducted if potential risk factors for test subjects have not been at least estimated beforehand in animal studies. The tedious and complex path leading from the discovery of active agents to approved potent drugs inevitably requires the use of animals, such as mouse models, at certain stages of scientific evaluation. The short life span of mice and the possibility of genetic manipulations significantly accelerate drug target identification and drug discovery. In addition, there is no ethical limitation to analyzing biosamples such as blood, urine, bile, liquor, and organ samples.

Despite undeniable differences between mice and man, genetically engineered mice and/or cells derived thereof to a certain extent can accurately model human diseases and can serve as tools for verifying the mechanisms of drug action. Nevertheless, a detailed understanding of the pathophysiological aspects in mice has contributed essentially to the development of new classes of drugs for the treatment of human disorders. For example, research in mouse models has considerably advanced the development of vaccines against poliomyelitis and amarillic typhus and novel therapeutic options in Alzheimer’s disease (Tau aggregation inhibitors), cystic fibrosis (CFTR activators), or HIV (Maraviroc).

**Fig. 3:**
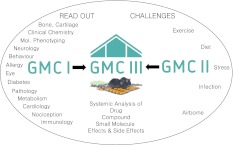
Systemic analysis of compounds and drugs in the German Mouse Clinic III

From drug and compound tests, a cornucopia of published datasets is available. Merging the multitude of datasets into models has proven tricky as these data derive from studies carried out under differing experimental conditions. Under the directive to gain a more comprehensive knowledge of the action of drugs, compounds, or small-molecule candidates, the concept of the German Mouse Clinic III recently emerged from GMC and GMCII as a novel third layer (Fig. 3). The GMCIII employs the scientific expertise and state-of-the-art technologies established in the GMC for standardized, high-throughout phenotyping of mouse mutants. In addition, the GMCIII makes use of tailor-made environmental challenges established within the GMCII, taking into account that the efficiency of drug therapy is modulated not exclusively by genes but also by environmental components.

The concept of the GMCIII is based upon the generation and integration of large-scale datasets, what we call “drug-types,” to enable a detailed spatial and temporal resolution of drug action. The approach promises to unravel molecular mechanisms and to advance medicine from an average to a more personalized level by phenotypic discrimination of responders from nonresponders, or by screening drugs in mouse models with different genetic backgrounds harboring the same mutation.

In Mouse200—a project within the GMCIII—acute and subchronic effects of the first-line antidiabetic drug Metformin were characterized in a widely used “standard” diabetes mouse model of monogenic obesity: the BKS-Leprdb mouse. In multiple disorders, combination therapy is favored due to drug resistance risk reduction and lowering of case-fatality ratios and therapy failure rates. Thus, we investigate the action of Metformin monotherapy and Metformin in combination with a novel drug in a large array of organs. In addition to assessing phenotypic “drug” characteristics in diabetic mice, detailed drug fingerprints in plasma, urine, feces, and an array of organs are generated by a joint approach of scientists from different disciplines applying the targeted and nontargeted technologies or metabolomics, proteomics, and transcriptomics. As a further facet in Mouse200, changes in the gut microbiome during diabetes therapy are evaluated. Large-scale datasets are expected to yield an estimated 100,000 data points for each individual mouse. Promising a substantial gain of knowledge, systems biology methods will be an integral part of the project facilitating the drug-type datasets for modeling dynamic, multidimensional processes accompanying drug exposure and for predicting potential interactions of drugs with pathways.

In summary, “systemic analysis of drug and compound action” in the GMCIII will be based upon the GMC scientific expertise and its comprehensive phenotyping technologies. The GMCIII will generate and integrate large-scale datasets to so-called “drug types” spanning a large array of medical areas. Such an approach will enable a detailed spatial and temporal resolution of drug action from the whole body, the organ, and down to the molecular level of what harbors the potential to discriminate responders from nonresponders. The concept of the GMCIII promises to unravel molecular mechanisms implicated in drug, compound, and small-molecule effects and side effects in mice and promises to advance medicine from an average to a more personalized level.

## Data handling and statistics

### Data management system

High-throughput mouse phenotyping requires sophisticated informatics systems to support coordination of the involved complex logistics as well as storage and analysis of the huge amount of data generated. In the German Mouse Clinic, custom integrated database-driven software called MausDB (Maier et al. 2008) has been developed for this purpose. MausDB meets four central requirements associated with a large-scale mouse phenotyping facility:

1. mouse husbandry (e.g., management of individual mice, cages and racks, matings, litters, pedigrees, and imports, as well as the management of housing costs, capacity planning, and monitoring of sanitary status)
2. logistics, scheduling of in-time cohort generation and of phenotyping experiments/pipelines
3. capture and structured storage of phenotyping result data, including metadata
4. (semiautomated) statistical analysis, visualization, and reporting of the results

MausDB integrates the free software environment for statistical computing [R (R Development Core Team 2010)] with its data management system. Accordingly, phenotyping data can be subjected to predefined R scripts from the MausDB user interface to generate high-quality diagrams and tables and standardized statistical analyses. The latest development is the integration of LaTeX—a free document preparation system—into the data-processing workflow. This allows the semiautomated generation of complete, individually composed phenotyping reports in pdf format with chapters for each screen, including diagrams, tables, and images.

MausDB is accessible for the scientific community through an open-source license (download from http://www.helmholtz-muenchen.de/ieg) and is already used in at least a dozen animal facilities worldwide.

### Statistical analysis

A major challenge in phenotyping mutant mouse lines is the statistical analysis of the dataset. Depending on the number of phenotyped animals and the kind of controls used (wild-type littermates or stock controls) as well as the kind of data and their distribution, different strategies may be appropriate for the analysis of the resulting raw data. While for the publication of data in many journals inferential statistics methods are desired, in other cases descriptive ways of analysis might be more suitable. Further possibilities for the discrimination of whether the mutant animals differ from the control cohort are provided by the use of reference ranges that can be established as soon as there are data from a sufficient number of control animals available. The use of reference ranges is restricted to large-scale projects where all animals are on the same genetic background and are examined under standard conditions.

The awareness about confounding factors brings further complexity into the processes of data analysis. To evaluate differences in genotype effects between males and females it is recommended that sex be included in the statistical model used, e.g., by using a two-way ANOVA. Many parameters are influenced by another factor that has to be taken into account for the analysis. For example, many parameters are affected by body mass or body size (e.g., fat and lean content, grip strength, locomotion and activity, bone mineral content, or heart weight). Earlier attempts to take these effects into consideration had been done by normalizing the data. We use integrated linear models to assess the effects of different covariates to refine the analysis for genotype-related effects (Kemter et al. 2010; Meyer et al. 2004, 2007; Schneider et al. 2006; Tocchetti et al. 2010). The multiple-testing issue also has to be addressed in large-scale phenotyping approaches where a series of parameters is determined from the same cohort of animals.

The next innovative step will be the implementation of more sophisticated analysis tools that do not focus on analyzing every phenotype variable on its own but try to follow an integrative approach. For example, Manhattan plots can also be applied to phenotype data (manuscript in preparation) in order to identify quickly hits in an overall variable overview. Dimension reduction methods are also promising tools and are currently being investigated for their ability to identify phenotypic deviations (manuscript in preparation).

Raw data of over 250 mutant mouse lines analyzed in first-line phenotypic characterization (which means more than 10 million data records) are stored in the GMC database. The first projects started to run meta-analysis and data-mining efforts on the complete data repository using results from all mutant lines. This approach requires the expertise of specialized bioinformatics research groups. The analysis of the complete dataset in the database has the potential to uncover previously unknown correlations between parameters and patterns associated with disease areas that might not be detectable through the analysis of single mutant lines alone. Even if these projects are still in the starting phases, the first study has already been published; it included expression profiling data of 90 organs from 46 mutant mouse lines. This analysis identified up to 232 differentially expressed genes in 45 organs (Horsch et al. 2008) and contributed to the identification of the recurring regulation of particular genes and groups of coexpressed genes.

Statistical approaches nowadays are important and indispensable tools for the analysis of phenotyping data. However, the complex interactions between parameters that contribute to a disease or syndrome cannot yet be completely simulated by software systems. For this reason, in the German Mouse Clinic an expert scientist evaluates the analysis for every phenotyping assay based on statistical results and his own experience and draws a final conclusion about the mutant line. Nevertheless, first attempts at understanding the relationships between the parameters for a simulation of biological systems and to support the scientist’s decision are begun.

## Technology development and imaging technologies

As the importance of mouse model systems for biomedical research gains more attention, the implementation of new phenotyping technologies also has to be further promoted. The development of innovative screening technologies stems from the need for more animal models for medical research. More experience is now available to know which kinds of tests were successful for the establishment of mouse models and which tests did not find clinically relevant phenotypes in the screened mutant mouse lines. Furthermore, there is now a phenotyping community large enough to form a critical mass of demand on the market. Therefore, it becomes more interesting for industry to operate this upcoming market for modern mouse phenotyping devices. In many cases, start-up companies collaborate with research institutes to discover what kind of experimental setup is needed for successful phenotyping. One benefit from the developments for the mouse phenotyping community is that there are established, standardized commercial systems available that permit the comparison of data across facilities. In addition, recent developments in phenotyping technologies are either directly derived from or more comparable to those for human diagnostics. However, there is still the need to invest some time in the direct development of new systems for which there are no commercial tools available yet. In the German Mouse Clinic both strategies are used in parallel: We invested in new commercial systems and established and implemented these new machines in our screening procedures. In addition, we collaborate with research institutes for imaging or physics to develop new phenotyping systems in a joint effort. Another option is to advance commercially available systems for use in large-scale mouse phenotyping approaches, e.g., magnet resonance technology is available for mice and small animals but the commercial systems are not yet ready for direct use in large-scale experiments.

The use of innovative technologies speeds up the process of phenotyping, which means that more mice can be analyzed in the same time or by fewer people. In some cases, innovative processes made it such that existing techniques could be implemented in large-scale, first-line screening procedures that had been restricted to use in second-line screens because of the long process. One example in the GMC is the use of micro-computed tomography (micro-CT), which is now ready to be implemented in the primary screening pipeline (and to replace DEXA technology for which machines are no longer manufactured). Scan time was decreased, and a boost in reconstruction speed in combination with the availability of automated functions for the analysis and quantification of the images permitted this step.

In addition to better speed in measuring and analysis and the possibility of automated analysis, the implementation of new and innovative techniques allows a higher accuracy of the obtained data. In most cases, this is also combined with a higher degree of standardization of the technique. For example, this is true of the Scheimpflug camera and optical coherence tomography (OCT) that replaced the slit lamp and ophthalmoscope for analysis of the anterior segment and the retinal fundus of the eye, respectively. Further examples of innovative test setups for first-line screening are summarized in Table 1.

**Table 1:** Innovative technologies for first-line screening that are or will be implemented in the phenotyping pipeline

Purpose	Innovative technology	Replaced technique
Analysis of the anterior part of the eye, e.g., anterior segment anomalies	Scheimpflug camera	Slit lamp analysis
Analysis of the retinal fundus	OCT	Ophthalmoscope
Functional analysis of the eye	Digitalized optokinetic drum	Optokinetic drum
Analysis of body composition	Minispec	DEXA
Analysis of bone mineral density	Micro computed tomography	DEXA
Analysis of cardiovascular function	Awake echocardiography	Blood pressure analysis
Analysis of lung function	FinePointe RC System	New application
Analysis of lung function	Pulmonary Function Testing System	New application
Analysis of skin permeability	Transepidermal Water Loss (TEWL)	New application

Imaging technologies are becoming more important for the characterization of mutant mice. The advantages of these modalities are that they are noninvasive, repeatedly applicable to the same animal, and therefore allow the monitoring of the development of a phenodeviation over time. One prominent example of an imaging technology in mouse phenotyping is magnetic resonance imaging. The German Mouse Clinic runs a nuclear magnetic resonance system equipped with a 9.4 Tesla magnet, several radiofrequency coils for different applications, and optimized gradient coils for great efficiency of in vivo scanning processes. In addition, it has auxiliary software and special accessories to facilitate investigation of mice. To improve the performance of the equipment, a cryogenic radiofrequency coil has been installed. The cryogenic coil delivers excellent resolution and soft tissue differentiation, providing, for example, very detailed anatomical images of the mouse cerebellum, cortical and subcortical structures, and brain vasculature. The NMR system is currently being used for anatomical analysis of mouse brains in projects involving neurodegenerative diseases and cerebral tumors. Imaging techniques are also being tested and improved for characterizing soft tissues present in the inner ear. The NMR system is being used to study morphological and metabolic changes in the liver in the context of diabetes research. Spectroscopic scanning is used to investigate specific fatty acid deposits in the mouse liver after a high-fat diet. This kind of analysis can be performed multiple times on the same animal to learn about the temporal changes of fat deposition in liver.

Further recent developments of new methodologies for secondary screening in the GMC are the analysis of breath gas, detection of volatile organic compounds (VOCs, manuscript in preparation), application of IR thermovision, and the use of hyperglycemic clamps in diabetes research. A summary of innovative technologies for secondary screening is given in Table 2.

**Table 2:** Innovative technologies for second-line screening

Name	Purpose
Magnet resonance tomography (MRT)	Imaging of
	Cardiac function
	Anatomical analysis of mouse brains
	Characterizing soft tissues present in the inner ear
	Study morphological and metabolic changes in liver
	Spectroscopic scanning to investigate fatty acid composition in the mouse liver
Breath gas analysis, detection of VOCs	Monitoring of exhaled volatile organic components for the analysis of metabolic alterations
IR thermovision	Analysis of temperature distribution on the body surface
Hyperglycemic clamp	Analysis of pancreatic β-cell function
Euglycemic-hyperinsulinemic clamp	Analysis of whole-body and tissue-specific insulin action
MicroCT and new software tools	Noninvasive measurement of abdominal body fat distribution
MicroCT and new software tools	Noninvasive measurement of liver fat content
IntelliCage	Automated analysis of aspects of cognitive function in group-housed mice

The implementation of innovative technologies is of the highest importance with respect to the welfare of the animals. With the 3R (reduction, refinement, replacement) directive in mind, innovative techniques contribute to the refinement of experiments, which in many cases is accompanied by the reduction of the number of experimental animals needed.

## Networking with international projects

The German Mouse Clinic is part of the international mouse phenotyping community and contributes to the success of many European and worldwide projects. Within EUMORPHIA (www.eumorphia.org; Brown et al. 2005), the phenotyping protocols were standardized across mouse clinics and phenotyping institutes, and a portfolio of tests (EMPReSS) was made accessible to the community. The GMC contributed to the phenotyping activities within EUMODIC (www.eumodic.org), where 500 mutant lines from the EUCOMM resource (www.knockoutmouse.org; Friedel et al. 2007) were analyzed in a joint effort. Furthermore, the GMC will be one of the partners in the IMPC (www.mousephenotype.org) and in the Infrafrontier Research Infrastructure (www.infrafrontier.eu).

### Infrafrontier: the European infrastructure for the phenotyping, archiving, and distribution of model mammalian genomes

Infrafrontier is a European initiative to build a sustainable research infrastructure for the systemic phenotyping, archiving, and distribution of mouse models for human diseases. It addresses three major challenges:

1. Building sufficient capacity for systemic phenotyping, archiving, and distribution of mouse models. These scientific platforms and services should be accessible for both individual research projects using mice of different genetic resources (knockouts, point mutations, complex genetics) and large-scale programs such as the International Mouse Phenotyping Consortium (IMPC)
2. Securing sustainable funding for the mouse production centers, mouse repositories, and primary phenotyping centers that contribute to the Infrafrontier Research infrastructure
3. Providing a single point of entry for the users of the scientific platforms and services offered by the Infrafrontier Research Infrastructure; underpinned by common quality standards and operating procedures with pan-European capacity and risk management.

The Infrafrontier consortium consists of 29 partners, representing the leading mouse clinics and archiving and distribution nodes, and related ministries and major funding bodies from 12 European countries and Canada. It is based on long-standing successful European joint efforts such as the EUMORPHIA and EUMODIC projects and European Mouse Mutant Archive (EMMA). Infrafrontier has been included in the European Strategy Forum on Research Infrastructures (ESFRI) Roadmap since 2006 and therefore is involved in a European policy process with the goals of (1) identifying novel research infrastructures of high scientific excellence and pan-European interest and (2) creating a European policy framework that facilitates the successful implementation of these research infrastructure initiatives.

The pan-European activities of the Infrafrontier Research Infrastructure will be coordinated by the Infrafrontier Legal Entity, which is currently being set up by the European member states. Through the EC-funded project InfraCoMP, Infrafrontier and IMPC coordinate their activities and formulate common strategies for mouse production, cryopreservation, and distribution, systemic phenotyping, access to phenotyping data, and community engagement.

### Relation to the European Mouse Mutant Archive (EMMA)

Even though the phenotyping data generated in the German Mouse Clinic are of remarkable value, the most synergistic effect will be achieved by simultaneously offering and providing mutant mouse lines easily and reliably to the scientific community.

All scientists using the phenotyping platform of the German Mouse Clinic have the opportunity to use the archiving services offered by EMMA (www.emmanet.org) (Hagn et al. 2007). EMMA is a nonprofit repository for the collection, archiving, and distribution of mouse mutant strains and plays a crucial role in exploiting the tremendous potential benefits to human health presented by the current research in mammalian genetics. Complemented with reproductive techniques (e.g., in vitro fertilization, embryo transfer), cryopreservation of germplasm and embryos provides a secure and economical way to maintain genetically unique mutant strains. Not only archiving but also speedy access to preserved mouse models is of major concern to the facilitation of scientific work.

To ensure consistent quality, cryopreservation, in vitro fertilization, and rederivation are performed according to EMMA standard operating protocols. Additionally, all processes follow standardized workflows, including several quality control steps, thereby ensuring a reliable archive. The long-term maintenance of cryopreserved samples is guaranteed by a backup storage system.

Phenotypes, and thus mutants of interest, can be identified by searching for the gene or the predicted phenotype in EuroPhenome (http://www.europhenome.org/), a project that provides access to raw and annotated mouse phenotyping data generated from primary pipelines such as EMPReSSslim and secondary procedures from specialized centers. Already more than 60 % of all mutant mouse lines phenotyped in the German Mouse Clinic and presented at EuroPhenome are available via EMMA. For all mutant mouse lines generated in large-scale projects such as EUCOMM, EUMODIC, and IMPC and phenotyped in the German Mouse Clinic, archiving and distribution via EMMA is mandatory.
